# Introduction to the special series, “The future of marine environmental monitoring and assessment”

**DOI:** 10.1002/ieam.4640

**Published:** 2022-06-09

**Authors:** Anders C. Erichsen, Anne L. Middelboe

**Affiliations:** ^1^ DHI A/S Copenhagen Denmark

**Keywords:** Acoustic monitoring, Automated sensors, Environmental modeling, Habitat modeling, Remote sensing

## Abstract

Traditional marine monitoring can be a resource‐intensive process that often covers a network of sampling stations where data are collected manually by divers, or discretely using in situ water samples at different depths at fixed positions followed by laboratory analysis. As such, environmental status is often reported after a delay of months or years. However, things are set to change for the better. Recent advances in technologies, such as remote sensing, machine learning techniques, modeling for non‐experts, acoustic monitoring, and intelligent integration of modeling and sensor measurements will revolutionize the future of marine environmental monitoring and monitoring systems. This special series touches upon some of the new technologies and models that may be an integrated part of ecosystem assessment and management in the future. Although technologies are being developed and integrated for marine monitoring around the world, the integration with ecosystem models is still in the early days. Still, this series highlights inspirational examples of the time ahead of us. *Integr Environ Assess Manag* 2022;18:888–891. © 2022 The Authors. *Integrated Environmental Assessment and Management* published by Wiley Periodicals LLC on behalf of Society of Environmental Toxicology & Chemistry (SETAC).

## Key Points

- Papers in the special series show advances in various technologies.

## INTRODUCTION

Seas and coasts are considered key drivers of the economy by the European Union (EU) and the Organisation for Economic Co‐operation and Development (OECD). Across Europe, compliance with national legislation, EU directives, such as the Water Framework Directive (2000/60/EC), Marine Strategy Framework Directive (2008/56/EC), and Habitats and Birds Directives (92/43/EEC and 2009/147/EC) and international conventions (supported by, e.g., Organisation for Economic Co‐operation and Development, 2016) for monitoring the status and development of the marine environment is central for the environmental management for governments and the private sector. This kind of monitoring focuses on a wide variety of biological, physical, and chemical parameters in the marine environment as well as critical pressures. While the exploitation of the sea is going on all over the planet and is expected to be an essential contributor to future economic development and human welfare, the UN Sustainable Development Goals (SDG no. 14: Life Below Water, being the most relevant here) claims that the sustainability of our oceans is under severe threat (United Nations [UN], 2021). However, today's monitoring programs are limited by high costs and are not optimally designed for a robust and proper assessment of the present ecological and/or environmental status and assessment of pressures. Efficient and time‐relevant monitoring methods are needed to understand the impacts and protect the sea—as the right balance between protection and exploitation is delicate and one of the most significant challenges for society today.

Today, all areas of the oceans are affected to some degree by human activities (Carstensen, 2014). At the same time, many monitoring programs worldwide are facing financial reductions, challenging our knowledge of the ecological status and the impacts of pressures. To support the ability to regulate using sustainability principles, impacts on marine ecosystems must be quantified, and precise knowledge of the baseline and potential impacts is vital. Otherwise, the growing human imprint on marine ecosystems may result in losses of ecosystems and the services they provide to human society (Carstensen, 2014); losses that might be irreversible, as discussed in Duarte et al. (2009).

Today, many marine monitoring programs consist of time and resource‐consuming elements largely based on ship‐based sampling and core sampling of benthic fauna by diver, or video surveys of flora and fauna to characterize the environmental conditions. Standardized procedures for sample analysis and quality control result in months or years of time‐lag reporting environmental status and trends. Advanced techniques are only applied to a limited extent, such as automated detection of seagrasses based on sailing and underwater drones and automated image analysis, online sensors and buoys for automated and online water chemistry measurements, and satellite observations for chlorophyll and vegetation cover. Some of the technologies have been subject to research for years, and especially, satellite ocean color estimates of chlorophyll *a* are regarded as effective tools to monitor the spatiotemporal distribution and variations of phytoplankton and primary productivity in the open ocean and coastal waters (D'Sa et al., 2011; Legaard & Thomas, 2006).

There are still unresolved issues to be addressed before the techniques can be used in operational monitoring, especially concerning methods for data integration, but as shown in Sengupta et al. (2020), different technologies can also strengthen and optimize other technologies or traditional monitoring, such as manually collected video transects.

However, the advances in known technologies, such as earth observations, drones, and sensors make it possible to develop an entirely new approach to marine monitoring in near real‐time. We suggest that approaches combining advanced modeling and technologies validated against traditional, thoroughly tested techniques, will guide sustainable marine management in the future by improving the knowledge base and predictability of management scenarios. This is in line with the conclusions of Skogen et al. (2021) that the way forward should be with models and observations in combination; to generate synergy and better support science, thereby increasing the knowledge and understanding of marine ecosystems.

Robust online monitoring systems, new digital technologies, and platforms are likely to revolutionize marine environmental monitoring. For example, recent developments within the environmental impact assessment (EIA) area aims to make advanced marine modeling, which is usually only accessible to experts, now easily accessible for nonexperts, and machine learning techniques are implemented in online services to benefit users (e.g., Huber et al., 2022). This technological development will, no doubt, continue in the future and will make it possible to quantify effects also in smaller and less‐complex projects.

In meteorology and forecasting of physical variables, advanced methods have been developed in recent decades to combine observations and models and increase the accuracy of assessment of the present conditions and of predictions. However, similar methods have not yet been developed to the same level for environmental variables, although establishing a precise and accurate ecological and/or environmental status would help analyze the potential for exploiting ecosystem services provided by the marine environment.

Nevertheless, ecosystem modeling has already been applied worldwide to analyze and manage marine ecosystems. It can be developed further into an efficient and advanced tool, enabling data interpolation from multiple sources in space and time by data assimilation. Data assimilation (DA) is a widespread practice used with waves and hydrodynamic (HD) models for hindcast analysis and global and regional forecasting. However, only a few models, such as Fennel et al. (2019), include biogeochemical variables because of the lack of suitable DA methods developed for nonlinear biogeochemical models (Gehlen et al., 2015). Currently, ecosystem models are capable of hindcasting, while it is challenging to develop real‐time model simulations and DA methods for real‐time data.

The present special series shows that the progress in combining observations and models into online monitoring systems is in its early development. These new technologies are being implemented within multiple sectors ranging from offshore wind to aquaculture and larger infrastructure projects. This special series consists of five papers describing a number of elements within monitoring programs, integrating new technologies, and to some extent, models examining several different taxonomic groups (phytoplankton, macroalgae and/or angiosperms, fish, and mammals).

The first paper (Lønborg et al., 2022) compares traditional methods and emerging remote sensing technologies to monitor submerged aquatic vegetation. Submerged aquatic vegetation (SAV) is a vital ecosystem element, as SAV influences the overall functioning of coastal ecosystems by (1) providing food, for example, for herbivorous waterbirds; (2) being shelter and hatching and nursery areas for many animals (e.g., fish); (3) improving water quality by absorbing nutrients; and (4) stabilizing sediments and decreasing HD energy, thus increasing sedimentation and water clarity (Lønborg et al., 2022). Hence, SAV is an ecosystem‐defining parameter and SAV supports biodiversity in many ecosystems. Both researchers and managers have investigated changes in SAV abundance and growth dynamics for decades to understand linkages to human pressures such as eutrophication. Submerged aquatic vegetation data have traditionally been supported by samples collected by divers and/or video transects, but this is time‐consuming, labor‐intensive, and gives a limited overview of the spatial coverage. Lønborg et al. (2022) conclude that a range of promising technologies exists for monitoring marine SAV. No technology is perfect—including the traditional diver and/or video transects—and Lønborg et al. (2022) suggest combining different technologies and increasing the use of machine learning (ML) models for postprocessing of data.

The second paper (Huber et al., 2022), continues along the lines suggested by Lønborg et al. (2022). It describes an SAV monitoring platform combining Sentinel‐2 satellite imagery and ML techniques for advanced mapping of SAV in Swedish nationwide marine waters. Submerged aquatic vegetation is in many locations sampled by video transects with kilometers between the transects, but combining, for example, video transect data with satellite data provides a method for extrapolating and providing spatial SAV coverage. Here, Huber et al. (2022) describe the feasibility and how new monitoring technologies and models (ML) can be applied in a cloud solution with the web interface, allowing nonmodelers to classify SAV based on, for example, traditionally collected diver and/or video transect data. The cloud solutions then use the classification to continuously train the ML model and eventually optimize the precision of the monitoring platform.

The third paper (Nezlin et al., 2022) analyzes if upwelling events may force local phytoplankton blooms in the Chesapeake Bay. Traditionally, phytoplankton—or chlorophyll *a*—is collected as discrete in situ water samples collected from different depths at fixed positions, and data used to assess, for example, the ecological status according to the Water Framework Directive (2000/60/EC). To assess if the upwelling forces phytoplankton blooms, Nezlin et al. (2022) apply Visible Infrared Imaging Suite (VIIRS) satellite data, as traditional phytoplankton data collection methods cannot be used in the assessment of bloom events, as neither sampling frequency nor spatial coverages (most likely) will capture those events and allow a robust analysis. In this paper, they conclude that a number of technologies exist for monitoring programs in freshwater and marine environments: Periodic research surveys, data collected at fixed stations, remotely sensed imagery, and numerical modeling. However, each approach has its limitations. Consequently, significant efforts aim to improve the quality, volume, and utility of data supporting ecological monitoring and assessment of pollution and eutrophication of economically important areas.

Macrander et al. (2022) evaluate the convergence of emerging technologies in the fourth paper looking at a risk‐based paradigm for marine mammal monitoring for offshore wind energy operations. In many marine areas, mammals are protected species and baseline monitoring is required post planning and construction of, for example, offshore wind energy operations. Visual inspections from boats or flights conduct traditional monitoring. In the paper, Macrander et al. (2022) discuss the innovative integration of near real‐time data streams from passive acoustic monitoring, habitat modeling, and visual observations, creating a tool to help prevent ship strikes on whales. Macrander et al. (2022) suggest the development of an adaptive regional risk assessment and risk management program. They recommend incorporating a broad spectrum of data from a number of monitoring platforms from multiple collaborators and innovative modeling as part of this program, allowing practitioners and stakeholders to move beyond the limits of existing standards.

The final paper (Bell et al., 2022) operates on a different scale. It discusses how sensor data and models can be applied to optimize aquaculture production and minimize the environmental impacts through frequent data sampling, coordination and sharing of data (an offshore and coastal marine aquaculture data network), and modeling. According to Bell et al. (2022), effective monitoring and forecasting are already the cases for some net‐pen salmon farming. Today, many aquaculture facilities face criticism as they can add to local eutrophication and impact the seabed below the cages. The authors are confident that automated sensors connected wirelessly to data stations, visualization tools, and acoustic and physical tagging technologies will soon be standard for monitoring ocean water and seafloor conditions and the behavior and health of farmed salmon.

Marine environmental monitoring systems are essential as they are the foundation of political decision‐making and administrative regulation concerning, for example, agriculture, aquaculture, industry, and marine spatial planning. Imagine if we were to design marine monitoring as was the case back in the early‐mid 80s, but with the technologies and models we have available today: Would we go for labor‐intensive snapshots as discrete water samples, diver transects, single fly‐over surveys, or would we argue that monthly or bi‐monthly measurement, transects with km‐wide gaps, and what can be seen from a flight over a day would not be representative of the status of the marine environment? We do not argue that the traditional methods do not provide any value but merely try to highlight the need for updating marine monitoring and acknowledging the value that new technologies provide, especially when integrated into combined products, for example, through modeling. Also, technologies create new opportunities, leading to research in yet‐unanticipated directions, as argued in Viollier et al. (2003).

Today, there is an increasing demand for data and transparency in decision‐making, and marine data must be detailed, precise, and readily available. Marine infrastructure projects, offshore energy, and food production are just a few areas where humans will impact marine ecosystems. However, when impacting, we need to protect—and improve—the marine ecosystems to the best of our ability, and here, we rely on new technologies and models to ensure that we optimize exploitation and protection.

## Data Availability

There are no data associated with this paper.

## References

[ieam4640-bib-0001] Bell, J. , Mandel, R. , Brainard, A. , & Wenning, R. (2022). Emerging monitoring practices in marine net pen aquaculture. Integrated Environmental Assessment and Management , *18*(4), 950‐963. 10.1002/ieam.4622 35438842

[ieam4640-bib-0002] Carstensen, J. (2014). Need for monitoring and maintaining sustainable marine ecosystem services. *Frontiers in Marine Science*, 1, 1–4. 10.3389/fmars.2014.00033

[ieam4640-bib-0003] Council Directive 92/43/EEC of 21 May 1992 on the conservation of natural habitats and of wild fauna and flora. EUR‐Lex‐31992L0043‐EN‐EUR‐Lex (europa.eu).

[ieam4640-bib-0004] Directive 2000/60/EC of the European Parliament and of the Council of 23 October 2000, establishing a framework for community action in the field of water policy. EUR‐Lex‐32000L0060‐EN‐EUR‐Lex (europa.eu).

[ieam4640-bib-0005] Directive 2008/56/EC of the European Parliament and of the Council of 17 June 2008, establishing a framework for community action in the field of marine environmental policy (Marine Strategy Framework Directive) (Text with EEA relevance). EUR‐Lex‐32008L0056‐EN‐EUR‐Lex (europa.eu).

[ieam4640-bib-0006] Directive 2009/147/EC of the European Parliament and of the Council of 30 November 2009 on the conservation of wild birds. EUR‐Lex‐32009L0147‐EN‐EUR‐Lex (europa.eu).

[ieam4640-bib-0007] D'Sa, E. J. , Roberts, H. , & Nabi Allahdadi, M. (2011). Suspended particulate matter dynamics along the Louisiana‐Texas coast from satellite observations. *The Proceedings of the Coastal Sediments 2011: In 3 volumes*. World Scientific, pp. 2390–2402. 10.1142/9789814355537_0179

[ieam4640-bib-0008] Duarte, C. M. , Conley, D. J. , Carstensen, J. , & Sánchez‐Camacho, M. (2009). Return to neverland: Shifting baselines affect eutrophication restoration targets. Estuaries and Coasts, 32, 29–36. 10.1007/s12237-008-9111-2

[ieam4640-bib-0009] Fennel, K. , Gehlen, M. , Brasseur, P. , Brown, C. W. , Ciavatta, S. , Cossarini, G. , Crise, A. , Edwards, C. A. , Ford, D. , Friedrichs, M. A. M. , Gregoire, M. , Jones, E. , Kim, H.‐C. , Lamouroux, J. , Murtugudde, R. , & Perruche, C. , The GODAE OceanView, Marine Ecosystem Analysis and Prediction Task Team . (2019). Advancing marine biogeochemical and ecosystem reanalyses and forecasts as tools for monitoring and managing ecosystem health. Frontiers in Marine Sciences, 6, 89.

[ieam4640-bib-0010] Gehlen, M. , Barciela, R. , Bertino, L. , Brasseur, P. , Butenschön, M. , Chai, F. , Crise, A. , Drillet, Y. , Ford, D. , Lavoie, D. , Lehodey, P. , Perruche, C. , Samuelsen, A. , & Simon, E. (2015). Building the capacity for forecasting marine biogeochemistry and ecosystems: Recent advances and future developments. Journal of Operational Oceanography, 8(Suppl 1), s168–s187.

[ieam4640-bib-0011] Huber, S. , Hansen, L. , Nielsen, L. , Rasmussen, M. , Sølvsteen, J. , Berglund, J. , Paz von Friesen, C. , Danbolt, M. , Envall, M. , Infantes, E. , & Moksnes, P. (2022). Novel approach for large‐scale monitoring of submerged aquatic vegetation—A nationwide example from Sweden. Integrated Environmental Assessment and Management , *18*(4), 909‐920. 10.1002/ieam.4493 PMC9290658 34270169

[ieam4640-bib-0012] Legaard, K. R. , & Thomas, A. C. (2006). Spatial patterns in seasonal and interannual variability of chlorophyll and sea surface temperature in the California current. Journal of Geophysical Research: Oceans , 111(C6), 1–21. 10.1029/2005JC003282

[ieam4640-bib-0013] Lønborg, C. , Thomasberger, A. , Stæhr, P. , Stockmarr, A. , Sengupta, S. , Rasmussen, M. , Nielsen, L. , Hansen, L. , & Timmermann, K. (2022). Submerged aquatic vegetation: Overview of monitoring techniques used for identification and determination of spatial distribution in coastal waters. Integrated Environmental Assessment and Management , *18*(4), 892‐908. 10.1002/ieam.4552 34750976

[ieam4640-bib-0014] Macrander, A. , Brzuzy, L. , Raghukumar, K. , Preziosi, D. , & Jones, C. (2022). Convergence of emeriging technologies: Development of a risk‐based paradigm for marine mammal monitoring for offshore wind energy operations. Integrated Environmental Assessment and Management , *18*(4), 939‐949. 10.1002/ieam.4532 PMC9299501 34617664

[ieam4640-bib-0015] Nezlin, N. , Testa, J. , Zheng, G. , & DiGiacomo, P. (2022). Satellite observations of phytoplankton growth associated with river discharge and wind‐driven upwelling in the Chesapeake Bay. Integrated Environmental Assessment and Management , *18*(4), 921‐938. 10.1002/ieam.4597 35218149

[ieam4640-bib-0016] Organisation for Economic Co‐operation and Development (OECD) . (2016). The ocean economy in 2030. 10.1787/9789264251724-en

[ieam4640-bib-0017] Sengupta, S. , Ersbøll, B. K. , & Stockmarr, A. (2020). Seagrassdetect: A novel method for the detection of seagrass from unlabelled underwater videos. Ecological Informatics, 57, 101083. 10.1016/j.ecoinf.2020.101083

[ieam4640-bib-0018] Skogen, M. D. , Ji, R. , Akimova, A. , Daewel, U. , Hansen, C. , Hjøllo, S. S. , van Leeuwen, S. M. , Maar, M. , Macias, D. , Mousing, E. A. , Almroth‐Rosell, E. , Sailley, S. F. , Spence, M. A. , Troost, T. A. , & van de Wolfshaar, K. (2021). Disclosing the truth: Are models better than observations? Marine Ecology Progress Series, 680, 7–13. 10.3354/meps13574

[ieam4640-bib-0019] United Nations (UN) . (2021). The Sustainable Development Goals Report. https://unstats.un.org/sdgs/report/2021/The‐Sustainable‐Development‐Goals‐Report‐2021.pdf

[ieam4640-bib-0020] Viollier, E. , Rabouille, C. , Apitz, S. E. , Breuer, E. , Chaillou, G. , Dedieu, K. , Furukawa, Y. , Grenz, C. , Hall, P. , Janssen, F. , Morford, J. L. , Poggiale, J. C. , Roberts, S. , Shimmield, T. , Taillefert, M. , TengberIgh, A. , Wenzhöfer, F. , & Witte, U. (2003). Benthic biogeochemistry: State of the art technologies and guidelines for the future of in situ survey. Journal of Experimental Marine Biology and Ecology, 285–286, 5–31.

